# An implementation evaluation of the Breaking Down human rights barriers to HIV services initiative in Ukraine

**DOI:** 10.1002/jia2.26328

**Published:** 2024-07-19

**Authors:** Diederik Lohman, Yevheniia Kononchuk, Alexandrina Iovita, Mikhail Golichenko, Valeria Rachinska, Pavlo Skala, Olga Gvozdetska, Serhii Myroniuk, Joseph J. Amon

**Affiliations:** ^1^ Consultant Maplewood New Jersey USA; ^2^ Consultant Kyiv Ukraine; ^3^ Community, Rights, Gender Department Global Fund Geneva Switzerland; ^4^ HIV Legal Network Toronto Ontario Canada; ^5^ 100% Life Kyiv Ukraine; ^6^ Alliance for Public Health Kyiv Ukraine; ^7^ Public Health Center Kyiv Ukraine; ^8^ Office of Global Health, Drexel University Dornsife School of Public Health Philadelphia Pennsylvania USA

**Keywords:** HIV, TB, human rights, stigma, discrimination, key populations

## Abstract

**Introduction:**

Globally, stark inequities exist in access to HIV treatment and prevention. The eastern European and central Asian region is experiencing the sharpest rise in new HIV acquisition and deaths in the world, with low rates of treatment and prevention services, especially for key and vulnerable populations who face a range of human rights‐related barriers to HIV prevention and treatment.

**Methods:**

An implementation learning evaluation approach was used to examine the implementation of the Breaking Down Barriers initiative targeting key and vulnerable populations in Ukraine. Between September 2022 and April 2023, researchers conducted 23 key informant interviews with individuals from the Ukrainian government, implementing organizations and human rights experts. Using a concurrent triangulation design, researchers and key informants, in a process of co‐creation, sought to describe programme accomplishments, challenges and innovations in implementation, between 2021 and 2023, including periods before and after Russia's February 2022 full‐scale invasion.

**Results:**

Eight rights‐based interventions related to HIV were identified in Global Fund programme documents and key informant interviews as making up the core of the Breaking Down Barriers initiative in Ukraine. These included programmes seeking to: eliminate stigma and discrimination; ensure the non‐discriminatory provision of medical care; promote rights‐based law enforcement practices; expand legal literacy (“know your rights”); increase access to justice; improve laws, regulations and policies; reduce gender discrimination, harmful gender norms and violence against women and girls; and mobilize communities for advocacy. These programmes received US$5.9 million in funding. Key informants reported that significant progress had been made addressing human rights barriers and scaling up interventions, both before and after Russia's invasion. Programme implementors adopted innovative approaches, including using paralegals, hotlines and other community‐led interventions, to ensure that key and vulnerable populations, including displaced individuals, were able to access prevention and care.

**Conclusions:**

An implementation learning evaluation approach examining programmes addressing human rights barriers to HIV services, designed as a process of co‐creation between researchers, programme implementors, government officials and human rights experts, can provide a robust assessment of programme outputs, outcomes and evidence of impact, despite a challenging operational environment.

## 1 INTRODUCTION

Since 2010, the eastern Europe and central Asian region has experienced the sharpest rise in new HIV acquisitions and deaths in the world, with a 49% increase in HIV acquisitions and a 46% increase in AIDS‐related deaths [1]. With an estimated 250,000 people living with HIV (PLHIV), Ukraine has the second largest HIV epidemic in the WHO European Region [2]. The epidemic is concentrated in key populations, with an HIV prevalence of 20.3% among people who inject drugs, 3.1% among female sex workers and 3.9% among men who have sex with men [2, 3, 4].

Russia's full‐scale invasion of Ukraine on 24 February 2022 profoundly changed all aspects of life for the country's 43 million people. More than 6 million people fled abroad, and millions more relocated internally [5, 6]. Many government services came to a halt, as did the economy [7]. The invasion had a major impact on Ukraine's delivery of healthcare: health facilities suddenly had to care for wounded soldiers and civilians; healthcare workers faced the difficult choice of staying or fleeing; health facilities were repurposed for non‐medical use; civilian patients in Russian occupied zones were forcibly evicted from health facilities or denied access to care; and medical supplies were requisitioned by Russian forces [8]. From the start of the invasion to the end of 2022, WHO recorded 790 attacks on health facilities [9]. By the end of 2023, the number of attacks had nearly doubled [10].

HIV programmes in Ukraine were significantly affected by the conflict [11, 12, 13]. PLHIV were displaced to areas of Ukraine that traditionally had low HIV prevalence and where the capacity of HIV services was insufficient [14]. Thousands of people were cut off from treatment and prevention services, including opioid substitution treatment (OST), which is banned in Russia [15, 16, 17, 18, 19, 20]. Transgender women faced challenges leaving the country if their identification documents did not match their gender identity [21].

Since 2017, as a part of the Breaking Down Barriers initiative of the Global Fund, Ukrainian organizations have implemented HIV‐related human rights programmes which seek to increase access to prevention and treatment services for key and vulnerable populations. The Breaking Down Barriers initiative provides technical and financial support for the implementation of rights‐based HIV, tuberculosis (TB) and malaria interventions in 24 countries. These include programmes aimed at addressing stigma and discrimination and ensuring people‐centred healthcare and law enforcement practices, as well as programmes aimed at mobilizing communities and increasing legal literacy and access to justice [22]. This implementation learning evaluation examined the implementation, outputs and outcomes of the Breaking Down Barriers initiative, focusing on the period between 2021 and 2023, before and after Russia's invasion, and in comparison with data from previous periods (2017‐2020).

## 2 METHODS

The evaluation of the Breaking Down Barriers initiative in Ukraine used an implementation learning approach drawing on a document review of programme monitoring documents alongside key informant interviews with implementers, government officials and human rights experts. While the assessment focused upon the scope, scale and quality of human rights programmes and their outcomes, as well as priorities for future investment, attention was also paid to national ownership, enabling environments and community‐led responses.

An implementation learning evaluation is an approach to multi‐organization assessment, typically incorporating qualitative and quantitative data related to implementation processes while also assessing changes in the broader context (such as the legal and policy environment). The approach seeks to facilitate quality improvement and identify recommendations for increased programme effectiveness. For our evaluation, we adapted the implementation learning evaluation principles outlined by Balasubramanian et al., focusing upon three steps: (1) gathering data on operational decision‐making by implementing organizations related to scale up of programmes; (2) collecting process and outcome data for each implementation objective; and (3) assessing multi‐level contextual factors that affected the implementation process, outputs and outcomes [23]. Two other domains related to our evaluation, programme quality and recommendations for future programme investment, are reported elsewhere [24].

Recognizing that conducting an evaluation of programme efforts amidst a war would be a challenge, we sought to orient the evaluation as a process of co‐creation with key informants [25]. Together, we pursued not only a greater understanding of implementation processes and outcomes, but, critically, the development of a consensus among implementors and government actors of priority areas for future investment. This approach also reflects efforts to decolonize and disrupt traditional evaluation research practices and reporting [26].

As a first step of the evaluation, three external evaluators (DL, YK and JJA) provided an overview of the objectives of the evaluation at a meeting convened by the Country Coordinating Mechanism (CCM) overseeing Global Fund activities in the country. Following the meeting, 23 programme implementers, government officials and independent human rights experts were identified as potential key informants, based upon recommendations from the CCM and from the leadership of individual implementing organizations participating in the Breaking Down Barriers initiative.

Potential key informants were provided with an explanation of the evaluation objectives and approach, and of the expectation of their involvement as experts and active participants in the evaluation process, helping to define key questions and contextualizing outputs, outcomes, facilitating factors and challenges in implementation. Consistent with the co‐creation approach, potential key informants were asked if they would consent to having their comments identified; however, they were also assured that they could provide comments without attribution.

All individuals who were asked agreed to participate and provided verbal consent, including individuals representing 16 different institutions: 100% Life, the Alliance for Public Health, the Public Health Center, Alliance Global, Ukrainian Network of People with Drug Dependence (VOLNA), Ukrainian Network of Women Who Use Drugs (VONA), FreeZone, Cohort, Legalife‐Ukraine, Ministry of Health, Ukrainian Prison Service, Hope & Trust, Ukrainian Helsinki Union for Human Rights, Ukrainian Legal Aid Foundation, Positive Women and TB People.

Semi‐structured interviews were conducted in‐person and remotely, using video, between September 2022 and April 2023. Interviews were conducted by fluent Ukrainian and Russian speakers, and lasted between 30 and 90 minutes. Documents reviewed included reports from programme implementers; Global Fund performance letters and briefing notes; national HIV strategy documents and human rights plans; specific key population or sector documents; UN Agency reports; donor implementation maps; integrated biobehavioural surveillance reports; and financial investment reports.

Researchers used a concurrent triangulation design to analyse information from the document review and key informant interviews in collaboration with key informants. Key results were first analysed broadly, according to the categories of intervention objectives defined by the Global Fund, examining the similarities and differences across different implementing organizations. Key challenges and facilitating factors were analysed using thematic data analysis approaches following Castleberry and Nolen [27]. Preliminary results were shared in several meetings with representatives from organizations participating in the evaluation. Feedback was incorporated in the preparation of the final results. Where information or observations regarding a specific intervention was provided by a single key informant, the informant is indicated, but, consistent with the objective of the co‐creation process, in most cases, challenges and outcomes were endorsed by multiple informants and by consensus in the feedback discussions. In these cases, no specific informants are indicated.

The protocol (2002007637) for the Breaking Down Barriers evaluation was reviewed by the Drexel University Office of Research's Institutional Review Board and, consistent with Center for Disease Control and National Institutes of Health guidance, was determined to be not human subjects research [28, 29].

## 3 RESULTS

Eight rights‐based HIV intervention objectives were identified in the Global Fund programme documents and by key informant interviews as the core of the Breaking Down Barriers initiative. These objectives included: (1) eliminating stigma and discrimination; (2) ensuring non‐discriminatory provision of medical care; (3) promoting rights‐based law enforcement practices; (4) expanding legal literacy (“know your rights”); (5) increasing access to justice; (6) improving laws, regulations and policies; (7) reducing gender discrimination, harmful gender norms and violence against women and girls; and (8) mobilizing communities for human rights advocacy. To support the implementation of activities addressing these objectives, Ukraine received US$5.9 million from the Global Fund. Of the eight objectives, the greatest funding was given to programmes addressing law enforcement practices and community mobilization and advocacy, and the least funding was provided to programmes reforming laws, regulations and policies (Table 1).

**Table 1:** Investments, implementing organizations and activities to remove human rights‐related barriers to HIV services by intervention objective (2021−2023)

Intervention objective	Investment	Implementing organizations	Activities
Eliminate stigma and discrimination	$ 390,272	100%, APH, PHC, AG, VONA, PW, KG, LU	Media campaigns to reduce HIV‐related stigma and discrimination. Training of journalists; raising awareness among religious communities about HIV and stigma towards key and vulnerable populations; stigma index studies
Ensure non‐discriminatory provision of medical care	$ 335,366	100%, PHC, APH, KG, H&T, LU	Trainings on HIV‐related stigma and discrimination for healthcare workers at all levels of care; hotlines; documentation systems
Promote human rights‐based law enforcement practices	$ 1,559,819	APH, LU, FZ	Training law enforcement about HIV prevention, harm reduction and substitution treatment
Expand legal literacy (“know your rights”)	$ 394,681	100%, APH, VOLNA, VONA, LU, TPU, AG, KG, FZ, PW, TU, ULAF, UHHRU, 100%	Peer paralegals empower community members to know and defend their rights; community training sessions; digital applications with information on HIV, including on rights and legal recourse
Increase access to justice	$ 798,428	100%, APH, VOLNA, VONA, LU, TPU, AG, KG, FZ, PW, TU, ULAF, UHHRU, H&T	Peer paralegals, community training sessions; digital applications; hotlines; referral to free legal aid services; human rights documentation systems
Improve laws, regulations and policies	$ 161,171	100%, APH, TPU	Fight for Health platform; human rights documentation systems
Reduce gender discrimination, harmful gender norms and violence against women and girls	$ 795,407	100%, APH, VOLNA, VONA, LU, TPU, AG, KG, FZ, PW, TU, ULAF, UHHRU	Gender‐specific programmes by women's and LGBT groups, paralegal programmes, hotlines, human rights documentation systems
Mobilize communities for human rights advocacy	$ 1,496,834	100%, APH, VOLNA, VONA, LU, TPU, AG, KG, FZ, PW, TU, ULAF, UHHRU	Paralegal programmes, human rights documentation, gender‐specific programmes
**Total**	**$ 5,931,978**		

Abbreviations: 100%, 100% Life; AG, Alliance Global; APH, Alliance for Public Health; FZ, FreeZone; KG, Cohort; LU, Legalife‐Ukraine; H&T, Hope and Trust; PHC, Public Health Center; PW, Positive Women; TPU, TB People Ukraine; TU, Teens Ukraine; UHHRU, Ukrainian Helsinki Human Rights Union; ULAF, Ukrainian Legal Aid Foundation; VOLNA, Ukrainian Network of People who Use Drugs; VONA, Ukrainian Network of Women who Use Drugs.

### 3.1 Eliminate stigma and discrimination

Ukraine has implemented a wide variety of programmes to address stigma and discrimination in multiple contexts, including in community, healthcare and law enforcement settings. For example, the Alliance for Public Health engaged journalists on drug policy issues, creating balanced narratives around people who use drugs. Implementing organizations used International Drug Users Day (1 November) and World AIDS Day (1 December) for awareness raising, and the release of the HIV Stigma Index drew attention to HIV stigma in the media and among health professionals. Key informants reported that these activities allowed for high‐profile, public, discussion of the human rights of PLHIV and key populations.

Key informants and programme reports also noted that a wide range of programmes aimed at reducing self‐stigma were implemented, including information sessions that combined “know‐your‐rights” information and discussion of access to justice with information on stigma and discrimination. For example, Legalife‐Ukraine, a community‐based organization of sex workers, organized trainings for 320 sex workers in 2020 and 551 sex workers in 2021 (Interview with Natalia Isayeva, Legalife‐Ukraine).

Other community organizations sought to address stigma and discrimination by creating new digital tools. FreeZone created the FreeLife app as a comprehensive information resource for current and former prisoners that included information on human rights and access to HIV prevention and treatment services. The application also allows users to file complaints and seek legal support in case their rights have been violated (Interview with Olga Karpenko and Nikolay Kukarkin, FreeZone).

Key informants discussed efforts to address the legal barriers that lead to stigma and discrimination. In particular, the parliamentary expert platform “Fight for Health” was identified as an important forum for the discussion of amendments to laws that criminalize HIV acquisition and exposure to HIV. The platform has also sought to codify access to testing, diagnosis, treatment, pre‐exposure and post‐exposure prophylaxis in Ukraine's healthcare legislation and HIV law, and to draft laws legalizing sex work [30]. Advocacy for these changes was initially suspended due to Russia's invasion but relaunched in the second half of 2022.

Key informants also described how community organizations have worked to raise awareness among religious communities and organizations to increase tolerance towards HIV‐vulnerable groups, targeting all major faith groups in Ukraine, including the Ukrainian Orthodox Church, the Ukrainian Greek Catholic Church, Muslim communities and Protestant churches [31].

### 3.2 Ensure non‐discriminatory provision of medical care

Annual programme reports indicated that, as a part of efforts to reduce discrimination in healthcare, 217 trainings of healthcare professionals were conducted, reaching 1652 nurses and 1582 medical doctors between 2020 and 2022. Key informants reported that one outcome of these efforts were their integration into routine trainings for health workers, including for primary care providers who following Ukraine's healthcare reform play a key role in providing care for key and vulnerable populations (Interview with Rachinska Valeria, 100% Life).

Similarly, in 2021, the Ukrainian government's Public Health Centre began integrating materials on human rights and medical ethics into continuing education programmes available on its website for healthcare providers. Materials related to patient‐centred care and stigma and discrimination were included in family medicine modules and material on community mobilization, communications and advocacy were included in its public health course. More than 135,000 users have received certificates for completing its online course on HIV, TB and key populations (Interview with Serhiy Myroniuk, Public Health Center).

Stigma and discrimination were also addressed in 100% Life Network's “Your Family Doctor” campaign which reached more than 2000 doctors between 2021 and 2023. The Alliance for Public Health conducted trainings of health workers on the provision of OST, expanding the number of trained healthcare workers from 177 to 460, and trained “transgender‐friendly” doctors and multidisciplinary teams serving transgender individuals in five regional centres (Interview with Pavlo Skala, Alliance for Public Health).

### 3.3 Promote rights‐based law enforcement practices

Interventions conducted by the Alliance for Public Health and Legalife‐Ukraine sought to reduce stigma and increase respect for human rights through work with law enforcement officials. For example, Legalife‐Ukraine trained 3872 law enforcement officers in 2020 on human rights (Interview with Natalia Isayeva, Legalife‐Ukraine). The Alliance for Public Health's work with law enforcement officials increased from small‐scale programmes in 2017 to trainings in 18 regions in 2020 and 21 regions in 2021. However, in 2022 following Russia's invasion, key informants noted that high turnover rates among police officers, many of whom joined the Ukrainian armed forces, posed a challenge, interrupting the continuity of programmes and requiring the development of new relationships of trust (Interview with Pavlo Skala, Alliance for Public Health).

### 3.4 Expand legal literacy (“know your rights”)

**Figure 1:**
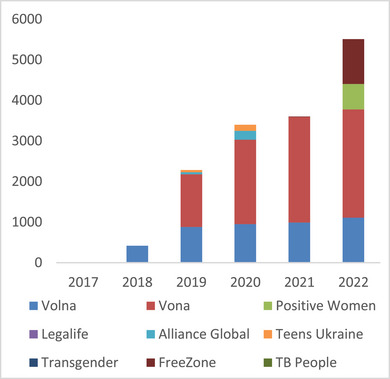
**Paralegal consultations by year**. Abbreviation: TB, tuberculosis.

A variety of approaches for improving legal literacy were implemented, including the use of traditional print materials, websites, phone apps, chatbots, telephone hotlines, informal legal literacy gatherings and sharing of rights information at health services providers. Figure 1 shows the dramatic increase in the number of individuals reached by paralegal programmes, run by nine different organizations and targeting different populations, between 2017, when no paralegal programmes existed, and 2022 when 5510 consultations took place.

**Figure 2:**
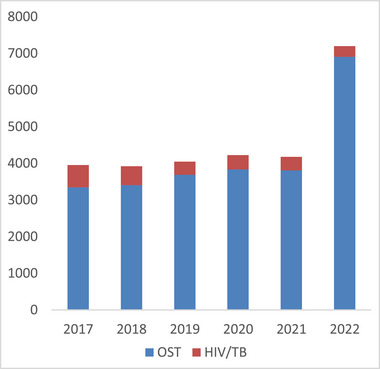
**Hotline consultations by year**. Abbreviations: OST, opioid substitution treatment; TB, tuberculosis.

By contrast, hotline consultations were largely stable between 2017 and 2021, until a surge in calls occurred after Russia's full‐scale invasion of Ukraine in 2022 (Figure 2). Hotlines provided a number of different services: they anonymously offer callers basic information about HIV, other sexually transmitted infections, and drug dependence; orient or link them to HIV or drug‐related services; and register and act on complaints about violations of their human rights.

### 3.5 Increase access to justice

**Figure 3:**
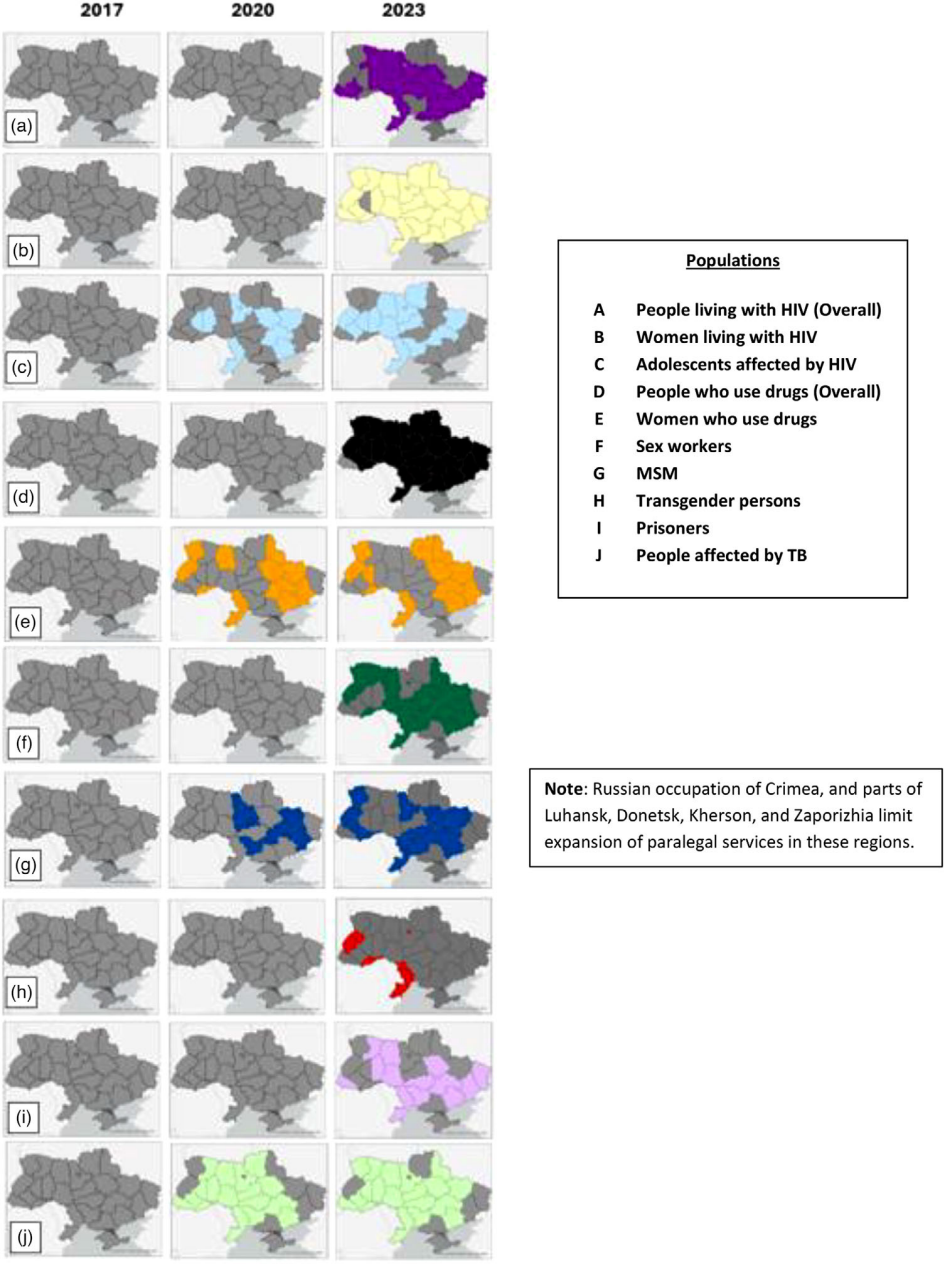
**Expansion of paralegal service by region and population (2017−2023)**. Abbreviations: MSM, men who have sex with men; TB, tuberculosis.

Once individuals are aware of their rights, access to justice programmes help people seek redress from rights violations. Between 2017 and 2023, paralegal programmes serving key and vulnerable populations dramatically increased their geographic coverage (Figure 3). Three of the paralegal networks (Legalife‐Ukraine, Positive Women and VONA) focused specifically on the needs of women, while FreeZone worked with individuals in detention. Datacheck, run by 100% Life and the REAct programme, run by the Alliance for Public Health, are similar to the paralegal programmes, working to monitor and respond to human rights violations at the community level, for example in the case of REAct, using “reactors,” social workers, project coordinators and other non‐governmental organization staff working with key and vulnerable populations, to provide information on rights, document violations and help provide redress. The REAct programme expanded from 11 regions in 2020 to 17 in 2022 (Interview with Pavlo Skala and Oksana Pashchuk, Alliance for Public Health). Both types of programmes reported high percentages of resolved claims, a key outcome, with 68% for 100% Life's Datacheck and 89% for ReACT in 2022.

### 3.6 Improve laws, regulations and policies

There was widespread agreement among key informants that community‐based or ‐led monitoring efforts to reform laws, policies and practices are among the most developed and impactful programmes addressing HIV in Ukraine. Between 2021 and 2023, key and vulnerable population communities prioritized both short‐ and long‐term advocacy goals. While Russia's full‐scale invasion of Ukraine negatively affected these programmes significantly, the European Union accession process created opportunities as Ukraine committed to align itself with European Union standards.

Advocacy also addressed implementation and policy challenges. For example, Volna used community‐led monitoring to prevent late delivery of OST and treatment interruptions, facilitating medicines procurement for 8125 patients. 100% Life and other civil society organizations advocated for greater state and local funding for the HIV response. Community‐based organizations used information campaigns, patient mobilization and direct advocacy with decision‐makers, and involved outside experts. This work significantly strengthened the role of communities and enhanced engagement with public authorities, which increasingly saw them as partners.

### 3.7 Reduce gender discrimination, harmful gender norms and violence against women and girls

Key informants reported increased geographic coverage of programmes addressing gender discrimination, including, for example, initiatives to ensure that transgender people and women who use drugs are able to access networks of “friendly doctors,” including gynaecologists, venereologists and infectious disease specialists. For example, regional coordinators of the organization “Positive Women” created a database of doctors who provide non‐discriminatory care.

Positive Women also engaged in advocacy with healthcare officials for new clinical guidance for pregnant women living with HIV. Vona's advocacy at regional levels resulted in the expansion of healthcare providers who are knowledgeable about, and friendly to, women who use drugs. Cohort advocated successfully for the establishment of a special service in Kyiv to provide transgender‐specific care.

Programmes to assist victims of gender‐based and domestic violence were also significantly expanded, especially in response to the COVID‐19 pandemic. Following Russia's invasion, key informants said that the need for gender‐based violence services has grown significantly, posing a new challenge for the government and civil society organizations and making linking to sexual and gender‐based violence services of humanitarian clusters a key challenge.

### 3.8 Mobilize communities for human rights advocacy

Ukrainian PLHIV organizations have a long history of mobilizing communities and conducting human rights advocacy. At the start of the Breaking Down Barriers initiative, key informants said that Global Fund principal recipients made a conscious choice to invest in community‐led organizations, including key population organizations that were less established. Key informants said that this strategy was showing results with dramatically expanded regional coverage. This allowed them to quickly and effectively respond to Russia's full‐scale invasion, linking significant numbers of people to access to HIV prevention and treatment services.

## 4 DISCUSSION

Before Russia's full‐scale invasion, Ukraine had made significant progress towards addressing stigma. Ukraine's PLHIV Stigma Index studies, conducted in 2010, 2013, 2016 and 2020, found consistent decreases in stigma over time for most indicators, including, for example, a decrease in “gossip” related to HIV status (from 30% in 2010 to 8% in 2020) and unauthorized disclosure of HIV status (from 37% in 2010 to 18% in 2020) [32]. Progress too was seen in attaining the UNAIDS 95‐95‐95 targets (representing the percentage of PLHIV knowing their HIV status, the percentage of those on sustained treatment and the percentage of those attaining viral suppression). These indicators were 75‐83‐94 in 2021 as compared to 56‐72‐89 in 2017 [33]. However, further progress towards these targets has been hampered by the Russian invasion and subsequent humanitarian crisis, which has also prevented further Stigma Index studies [34]. Responding to the challenges of Russia's invasion, programme implementers took actions to strengthen the resiliency of programmes; ensure greater engagement of key and vulnerable populations to identify and defend human rights issues; and took advantage of opportunities to conduct advocacy for legal and policy change.

### 4.1 Resiliency of programmes

Evidence from the 2022 to 2023 assessment found numerous examples of resiliency in the implementation and scale up of rights‐based HIV programmes. The number of mechanisms for key and vulnerable populations to seek access to remedies has dramatically increased despite the war. Paralegals, hotline operators and owners of mobile applications have all been trained to provide primary legal aid to clients, and to refer them to professionals if secondary or tertiary legal aid is required. Efforts to link community human rights mechanisms to Ukraine's free legal aid services have secured the eligibility of key and vulnerable populations and focus on training of legal aid coordinators.

### 4.2 Readiness to demand and defend rights

Thousands of members of key and vulnerable populations have reported violations of their rights to community‐led monitoring and documentation initiatives, demonstrating both an awareness of their rights and a willingness to report violations. Moreover, since the Russian invasion, the number of inquiries and complaints from key and vulnerable populations have significantly increased, forcing, among others, the OST and Drug Dependence Hotline to increase their operating hours, and establish new channels of communication to meet the demand for information and help ensure clients’ continued access to health services.

### 4.3 Improving the legal and political environment

Significant improvements in the legal environment for key and vulnerable populations facilitated the implementation of all intervention programmes. However, the single most significant legal barrier, the criminalization of people who use drugs, and aspects of sex work, persists. Nonetheless, improvements to the legal environment included: changes to the HIV Law (2023); changes to regulations for take‐home doses of OST (2022); the removal of legal provisions banning men who have sex with men from becoming blood donors (2021); and the decision to transfer the prison healthcare system from the Ministry of Justice to the Ministry of Health (2021).

Overall, these legal changes have improved access to treatment for PLHIV and people who use drugs by removing discriminatory legal provisions and reducing HIV‐related stigma and discrimination. Several further amendments to key laws affecting key and vulnerable populations are currently pending before Ukraine's parliament. These amendments, including the decriminalization of transmission of HIV and the recognition of same‐sex partnership, were drafted with active participation of community organizations through the Parliamentary Platform.

Several limitations should be recognized. First, conducting an evaluation amidst a war is a challenging process. Russia's attacks on Ukraine's electrical grid repeatedly disrupted interviews. Many key informants faced emergencies related to the war that took precedence over interviews with the evaluation team. Second, the evaluation relied significantly upon programme reports and the perspectives of programme implementers. Given the ongoing war, it was not feasible to include the perspectives of programme beneficiaries.

Despite these limitations, the implementation learning evaluation method, using a co‐creation approach, engaged key stakeholders, who had long‐term experience and perspective in the implementation of diverse rights‐based programmes, and who felt ownership in the evaluation process and its ultimate goal of contributing to decisions on future investment.

## 5 CONCLUSIONS

The results of our evaluation found that Ukrainian organizations were successfully scaling up and adapting programmes addressing stigma and discrimination in communities and health settings; working to address harmful laws and police practices and increase legal literacy and access to justice; reducing gender discrimination and violence; and mobilizing communities affected. Ukrainian organizations responded resiliently to challenges, finding innovative solutions to reach those displaced and to ensure the continuity of HIV programmes.

## COMPETING INTERESTS

The Alliance for Public Health provided a financial grant to the IAS within the framework of a Global Fund regional grant for the preparation of this special issue. No representatives of the IAS contributed to the preparation or review of the article. The authors declare no competing interests.

## AUTHORS’ CONTRIBUTIONS

JJA, DL and YK designed the evaluation approach. DL and YK conducted the field research in collaboration with key participants. VR, PS and SM were key informants. All authors contributed to the data analysis. JJA and DL wrote the first draft of the manuscript. All authors contributed to subsequent revisions and approved the final submission.

## FUNDING

Funding for the evaluations in 2019−2020 and 2021−2023 were provided by the Global Fund in a grant to Drexel University.

## Data Availability

Additional data are available in the full evaluation report: https://www.theglobalfund.org/en/throughout‐the‐cycle/community‐rights‐gender/
